# Health Tract No. 340

**Published:** 1868-07

**Authors:** 


HEALTH TRACT NO. 340.

### NON SEQUITUR

Literally means "it does not follow"; it is a disappointment to the hearer; and disappointments may be so intense, or so numerous, as to make a man sick, hence the title may be lugged in as a text for a health tract; and a respectably sized book might be made on the subject; we will condense it into a page. A great name, made famous first by a distillery dream, was announced to "speak a piece" on Luther and Calvin; he forgot all about it and the piece was'nt spoken. Another announcement was made and the "piece was spoke," proving very satisfactorily by the time that nine-tenths of it was delivered, the personality of the devil; from which two conclusions were drawn; first, that any senator not voting for the removal of the president of the United States, would condemn himself to everlasting infamy. Second, that the negroe was entitled to vote, as well as any other white man; this was Thursday May 7th, 1868, at 8 P. M. 46th St, New York; on the Sunday night following, on Broadway New York, there was the anniversary of the American Missionary Society; the sermon of the occasion was brought to three focusses or "foci" according to "all Roman Story.' 1st, The President ought to be officially guillotined; 2nd, Everybody ought to be allowed to vote; 3d, The whole world, including "the South" ought to be reconstructed. These are political non sequiturs, or false pretences. Opening a Health Journal to day, we read "Caste in Philadelphia," wherein the venerable editor begins to prove that Bostonians are Idolators; that the god of their Idolatry is "The Almighty Dollar;" that if a man has been to the states prison and only brings a pile big enough to the "Hub,"

"Its Ticket good enough to pass
"Him readily through every door."

So much the better we should say for the man who has the ticket! The next point he proves is "Philadelphia worships family;" consequently, was as much a pro-slavery city as Charleston or New Orleans, and would'nt let "cullud pussons" ride in the cars, because the "old boy" was black and had too large a family; old enough, being one of the first settlers, but had rather a promiscuous set about him.

On another page our antediluvian friend, a certain company having sent him a lot of tea, declares that tea is "diuretic," and excellent; that the editor of the Congregationalist and Recorder says so; and that having used tea all his life and not having been sick for thirty years, he commends it to his readers as a healthy beverage; we consider this the biggest non sequitur—and "whopper," of them all; the sequitur absolute being, that it would be "excellent" for the whole human family tc be put through a course of diuretics by tea drinking.

